# Comparison of the Long-Term Outcomes of Endoscopic Papillary Large Balloon Dilation Alone versus Endoscopic Sphincterotomy for Removal of Bile Duct Stones

**DOI:** 10.1155/2018/6430701

**Published:** 2018-07-02

**Authors:** Tao Li, Jun Wen, Like Bie, Biao Gong

**Affiliations:** Digestive Endoscopy Center, Department of Gastroenterology, Ruijin Hospital, Affiliated to Shanghai Jiao Tong University School of Medicine, Shanghai, China

## Abstract

**Background and Aims:**

Endoscopic papillary large balloon dilation (EPLBD) alone is an alternative to endoscopic sphincterotomy (EST) for treatment of common bile duct (CBD) stones. However, limited data exists regarding comparison of the long-term outcomes for these techniques. In this study, we compared the long-term outcomes after EST with those after EPLBD alone for removal of CBD stones.

**Methods:**

The records of patients with EST or EPLBD alone referred for CBD stones retrieval between June 2008 and July 2015 were retrospectively reviewed. Complete stone clearance, ERCP-related adverse events, and late biliary complications during long-term follow-up were analyzed.

**Results:**

Basic patient characteristics were similar between the groups that underwent EST (*n* = 60) and EPLBD alone (*n* = 161). EPLBD compared with EST resulted in similar outcomes in terms of complete stone clearance (99.4% versus 100%, *P* = 0.54) and ERCP-related adverse events (6.8% versus 6.7%, *P* = 1.00). The mean duration of the follow-up was 74.5 months and 71.6 months who underwent EST and EPLBD alone, respectively (*P* = 0.42). Late biliary complications were occurred frequently in the EST group than in the EPLBD alone group (11 [18.6%] versus 16 [10.2%]), although the difference did not reach statistical significance (*P* = 0.11). Multivariate analysis showed that mechanical lithotripsy ([OR], 2.815; 95% CI, 1.148–6.902; *P* = 0.024) was significantly associated with late biliary complications.

**Conclusion:**

As an alternative to EST, EPLBD has similar efficacy and safety for managing CBD stones. During long-term follow-up, patients who underwent EPLBD alone may have fewer late biliary complications compared with those after EST. In addition, mechanical lithotripsy may be an independent risk factor for late biliary complications.

## 1. Introduction

Endoscopic sphincterotomy (EST) is a well-established standard method for extraction of common bile duct (CBD) stones. However, adverse events such as bleeding, perforation, pancreatitis, and cholangitis occur in 5% to 10% of patients who underwent EST [1–3]. Furthermore, EST causes a permanent reduction in biliary sphincter function and results in additional late biliary complications [4]. To remedy this disadvantage, the technique of endoscopic papillary large balloon dilatation (EPLBD) using a balloon larger than 12 mm was introduced for removal of large CBD stones [5, 6]. Since then, several studies have reported that EPLBD with or without EST produced satisfactory results with large CBD stone clearance without an increased risk of severely adverse events [7–11]. In addition, some authors also showed that the short-term therapeutic outcomes and complications related to EPLBD for treatment of large bile duct stones are comparable to those after EST [12]. However, up to now, there is no consensus yet on the long-term efficacy and safety of EPLBD alone compared with those after EST for treatment of CBD stones. Therefore, in this study, we aimed to compare the long-term outcomes between EPLBD alone and EST for the management of CBD stones. In addition, risk factors associated with late biliary complications were also analyzed.

## 2. Materials and Methods

### 2.1. Patients

The study was conducted based on data recorded in a prospectively maintained registry of patients who underwent EPLBD alone or EST referred for bile duct stone retrieval between June 2008 and July 2015 in our center. Patients were retrospectively selected based on the following inclusion criteria: [1] age over 18 years, [2] a large or multiple CBD stones (≥10 mm or ≥3 stones), [3] CBD stones visualized during endoscopic retrograde cholangiopancreatography (ERCP). However, patients were excluded if they met the following criteria: [1] concomitant intrahepatic stones or pancreaticobiliary malignancies, [2] distal CBD with stricture, [3], coagulation disorders (such as international normalized ratio [INR] > 1.5 and platelet count < 50,000/mL). Patients' clinical information, including demographic characteristics, present and past medical history, laboratory and imaging results, ERCP procedures, and related adverse events were collected and analyzed. Analysis of these data was approved by the institutional review board.

### 2.2. Endoscopic Procedures

**Figure 1:**
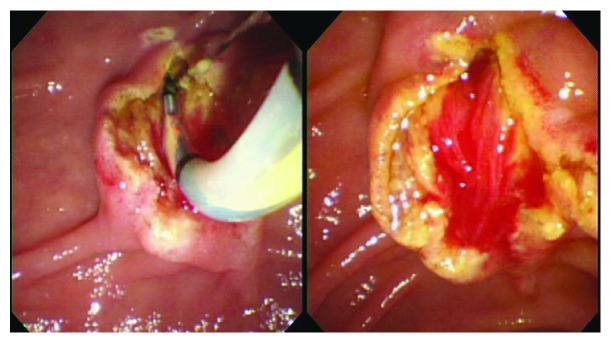
Complete endoscopic sphincterotomy was performed.

**Figure 2:**
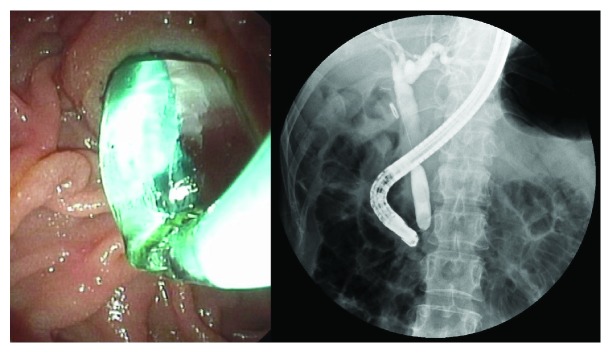
Endoscopic papillary large balloon dilation alone was performed.

After communicating with the patients, written informed consent for the endoscopic procedure was signed from all patients. ERCP was performed using a side-viewing endoscope (TJF-240, JF-260V; Olympus Co. Ltd., Tokyo, Japan) by two experienced endoscopists (Biao Gong and Like Bie). When selective cannulation was achieved, a diagnostic cholangiogram was performed to assess the size and number of bile duct stones, the diameter of CBD. Either EST or EPLBD alone was chosen at discretion of the endoscopists to enlarge the bile duct opening of the duodenum. The choice of methods depended on the preference of the endoscopist in most cases. In addition, we preferred EPLBD to EST in the following cases: (1) the presence of periampullary diverticulum and (2) patients with surgically altered anatomy. But when the distal bile duct was not sufficiently dilated for some patients, excessive balloon dilation beyond the diameter of the distal bile duct may increase the risk of perforation; therefore, we selected the EST. For patients in the EST group, a complete sphincterotomy was performed with a pull-type sphincterotome and the sphincter was divided up to the transverse duodenal fold. A complete sphincterotomy was defined by the free passage of a fully bowed sphincterotome and the presence of spontaneous bile drainage (Figure 1). For patients in the EPLBD alone group, a large-size balloon dilator ≥ 12 mm (CRE wire-guided dilator, Boston Scientific, MA, USA) was passed over a prepositioned guidewire, positioning the deflated balloon across the main duodenal papilla. The size of the balloon was chosen according to the size of the bile duct, the papillary orifice, and the largest CBD stone, but it did not exceed the diameter of the distal CBD. The balloon was then gradually filled with diluted contrast medium and remained inflated until the waist of the balloon had disappeared on fluoroscopy. Subsequently, the fully expanded balloon was maintained in position for 60 seconds. EPLBD alone was defined as dilation of major duodenal papilla and distal CBD endoscopically using a balloon dilation catheter of diameter from 12 to 20 mm to facilitate the removal of difficult bile duct stones without any EST (Figure 2). Besides, the maximum diameter of the balloon used was no more than 15 mm in our center. The stones were retrieved from the bile duct with a basket or a retrieval balloon. Mechanical lithotripsy (ML, Lithotriptoren; MTW Endoskopie, Germany) was used to crush the stones if needed. After complete stone removal, an occlusion cholangiogram was obtained in the end of the procedure. Prophylactic antibiotics were not routinely administered before ERCP.

### 2.3. Evaluation of Clinical and Endoscopic Data

Complete stone clearance was defined as the absence of filling defects on occlusion cholangiogram. Overall success rate was defined as the rate of complete CBD stone retrieval irrespective of the use of ML or number of ERCP sessions. Adverse events after ERCP were sorted and graded according to the consensus guidelines [13, 14].

### 2.4. Measurements during Follow-Up

All the patients were followed up periodically after complete stone clearance. During the follow-up, a blood sample for liver function tests were scheduled at every 6 months and the abdominal ultrasonography was checked at least once yearly after removal of CBD stones. If the patient was unable to visit outpatient clinic, the patients' primary follow-up information was contacted by telephone. If clinical symptoms are indicative of biliary complications, such as stone recurrence and cholangitis, additional workups, such as imaging modalities (computed tomography (CT) or magnetic resonance cholangiopancreatography (MRCP)) and/or ERCP, were performed to confirm them. Late biliary complications occurring at least 30 days after complete stone removal were classified by symptoms, laboratory data, and imaging findings. Stone recurrence was defined as a newly detected CBD stone by cholangiography after the determination of complete stone removal. Cholangitis was diagnosed when patients had at least three of the following: pain, fever, jaundice, and elevation of hepatobiliary enzyme levels.

### 2.5. Statistical Analysis

Baseline patient characteristics and basic outcome variables are presented as means and standard deviations (SD) for continuous variables or a frequency and a percentage for categorical variables. Statistical analysis was performed by using the chi-squared test or the Fisher exact test for noncontinuous variables and the Student *t*-test for continuous variables. The distributions of the occurrence of late biliary complications over time were estimated by the Kaplan-Meier product moment method, and the log-rank test was used to assess differences between the groups. To evaluate the risk factors for late biliary complications, potential risk factors were initially assessed by univariate analysis. The predictive risk factors with a *P* value < 0.2 in univariate analysis were then included in a multivariate logistic regression analysis. *P* values of <0.05 was considered to be statistically significant. Data analyses were performed using the IBM SPSS Statistics for Windows (version 20.0; IBM Corp, Armonk, New York, USA).

## 3. Results

### 3.1. Patient Demographics

Database analysis identified 221 patients referred for CBD stone retrieval during the study period; 60 patients were enrolled in the EST group, and 161 patients were enrolled in the EPLBD alone group. Patients' characteristics are summarized in Table 1. There were no between-group differences in patient demographic details, number and size of CBD stones, and CBD diameter. However, the presence of the periampullary diverticulum was slightly different in the two groups. 29 (48.3%) patients from the EST group and 67 (41.6%) patients from the EPLBD alone group had undergone a previous cholecystectomy (*P* = 0.45). Cholecystectomy was performed within 1 month after EST in 7 patients (of 14 with gallbladder in situ with stones) and in 14 patients (of 49 with gallbladder in situ with stones) after EPLBD alone. No significant difference was observed in the gallbladder status between the two groups.

### 3.2. Immediate Treatment Outcome

The overall complete stone clearance rate was 100% (60/60) in the EST group and 99.4% (160/161) in the EPLBD alone group (*P* = 0.54) (Table 2). The causes of failure in one patient were larger stone impaction and difficulty in removing it, and the patient underwent surgery in the end. A total of 98.3% of patients in the EST group and 98.8% of patients in the EPLBD alone group had stones cleared in the first session (*P* = 0.81), respectively. The use of ML was more frequently in the EST group than in the EPLBD alone group (17 [28.3%] versus 29 [18%]), although the difference did not reach statistical significance (*P* = 0.10). In addition, there is no difference in the mean length of hospital stay between the EST and EPLBD alone groups (7.4 versus 7.8, *P* = 0.49).

### 3.3. ERCP-Related Adverse Events

ERCP-related adverse events occurred in 6.7% of patients in the EST group and 6.8% of patients in the EPLBD alone group, respectively. The details are shown in Table 3. The incidence of post-ERCP pancreatitis (3.3% versus 5.0%, *P* = 0.73) and cholangitis (3.3% versus 1.9%, *P* = 0.62) were not significantly different between the EST and EPLBD alone groups. All of these patients improved with conservative management. Severe complications, including perforation, bleeding, and severe pancreatitis did not develop in any patients. None of the patients died after ERCP procedures.

### 3.4. Outcomes of the Long-Term Follow-Up

**Figure 3:**
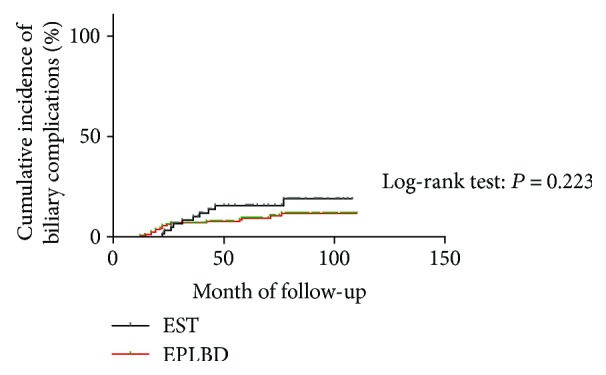
Cumulative incidence of late biliary complications. The red line indicates the EPLBD group, and the black line corresponds to the EST group. Kaplan-Meier estimates of the proportion of patients with late biliary complications in the endoscopic sphincterotomy (EST) (*n* = 59) and endoscopic papillary large balloon dilation alone (EPLBD) (*n* = 157) groups (*P* = 0.223 by log-rank test).

Follow-up data was collected every year after complete stone clearance, and final updates were done in October 2017. Five patients were lost during the follow-up period. Six patients died during the follow-up period. The causes of death were not related to biliary complications. No significant difference was observed between the EST and EPLBD alone groups in the mean duration of follow-up from the end of complete stone clearance to the last observation or death (74.5 ± 20.2 versus 71.6 ± 24.5 months, respectively, *P* = 0.42) (Table 4). The incidence of late biliary complications occurred frequently in the EST group than in the EPLBD alone group (11 [18.6%] versus 16 [10.2%]), although the difference did not reach statistical significance (*P* = 0.11). Kaplan-Meier analysis also revealed no difference of the incidence of late biliary complications between the EST and EPLBD groups (log-rank test, *P* = 0.223) (Figure 3). Stone recurrence were observed in 16 and 8 patients for the EPLBD alone and EST groups (10.2% versus 13.6%, *P* = 0.47), respectively. All recurrent stones were successfully extracted by ERCP procedure again. In addition, a significantly higher incidence of cholangitis without stone recurrence was observed in the EST group than in the EPLBD alone group (3 [5.1%] versus 0 [0%]; *P* = 0.02). These patients were treated with antibiotics, and their symptoms resolved in a few days.

### 3.5. Risk Factors for Late Biliary Complications

To examine predictive risk factors for late biliary complications, potential risk factors of patient characteristics and ERCP procedures were compared between the late biliary complication group (*n* = 26) and without late biliary complication group (*n* = 190). ML was identified as a risk factor for late biliary complication based on univariate analysis. The multivariate analysis also showed that ML ([OR], 2.815; 95% CI, 1.148–6.902; *P* = 0.024) was significantly associated with late biliary complications (Table 5).

## 4. Discussion

EST is the most commonly used method as a standard technique for the treatment of CBD stones [13]. However, EST has many early complications including hemorrhage, cholangitis, and perforation and late complications associated with dysfunction of the sphincter [15, 16]. To overcome this disadvantage, EPLBD with or without a preceding EST was introduced for removal of larger bile duct stones [5, 6]. In addition, EPLBD is especially suitable for patients with an unfavorable anatomy for EST, such as for patients who have underwent Roux-en-Y or Billroth II gastrectomy [17–19].

Periampullary diverticulum is known to be associated with an increased frequency of pancreatobiliary diseases, because the ampullary area in patients with periampullary diverticula is composed of thin mucosa without sphincter muscle. Besides, the periampullary diverticulum tends to distort the anatomy of the duodenum and the sphincter. So this altered state makes EST more difficult and dangerous for these patients [2, 20]. So in our present study, we preferred EPLBD alone in patients with periampullary diverticulum.

In our study, both EST and EPLBD alone for management of bile duct stones resulted in similar outcomes with respect to complete stone clearance (100% versus 99.4%), which is similar to previous results regarding overall stone clearance [12, 21, 22]. In addition, ML might be used more often in the EST group compared to the EPLBD group for removal of large or multiple stones in our study (17 [28.3%] versus 29 [18%]). Our results were consistent with previous studies [21, 23, 24], because the widened ampullary orifice made by EPLBD alone facilitates easier extraction of relatively large bile duct stones and may reduce the need for ML compared to EST.

Major adverse events typically related to both EST and EPLBD are pancreatitis, bleeding, and perforation. In the current study, ERCP-related adverse events were similar between the two groups (6.7% versus 6.8%, *P* = 1.00). With respect to the post-ERCP pancreatitis, many concerns have been raised about pancreatitis after balloon dilation with increasing balloon size. However, in this study, the risk of post-ERCP pancreatitis was not increased with EPLBD compared to EST (5.0% versus 3.3%). Recently, some studies also reported that the increase in balloon size does not affect the development of pancreatitis after EPLBD for bile duct stone removal [9, 25]. Based on these results, post-ERCP pancreatitis associated with EPLBD may occur at a lower incidence rate and with a similar incidence compared to EST. Furthermore, although severe complications did not develop in our study, it should be mentioned that EPLBD may cause very serious bleeding and perforation [26, 27]. The most likely explanation would be that large-diameter balloon dilation would tear the ducts and result in bleeding and perforation.

Several studies have investigated the long-term outcomes of EST. The incidence of biliary complications such as stone recurrence and cholangitis was reported in 9.2% to 17.7% of the patients which is similar to ours [28–31]. Park et al. [8] in a retrospective study of EPLBD alone for the treatment of large CBD stones reported that the incidence of biliary complications such as stone recurrence and cholangitis was 16.8% during a mean follow-up period of 1398 days. However, up to now, few studies have investigated comparison of the long-term outcomes of EPLBD alone versus EST for removal of bile duct stones. In our present study, the median duration of the follow-up from the end of the complete stone clearance to the last observation or death was 74.5 months and 71.6 months who underwent EST and EPLBD alone, respectively (*P* = 0.42), which we believe was sufficient to determine the incidence of late biliary complications. In the current study, the incidence of stone recurrence in the EPLBD alone group (10.2%) was not significantly different from the EST group (13.6%) which is similar to previous studies [4, 8, 32–34]. As far as we know, several studies have demonstrated a severe permanent impairment of sphincter of Oddi function after EST; subsequent duodenobiliary reflux and bacterial contamination after EST can cause late biliary complications such as cholangitis and stone formation [4, 35]. However, EPLBD used large-diameter balloon (≥12 mm); extensive dilation of the sphincter may lead to the tearing of the sphincter muscles, and the degree of papillary damage would be more serious in EPLBD compared to EPBD. Recently, a prospective randomized study revealed that EPLBD alone resulted in persistent and comparable loss of sphincter of Oddi function [36]. Therefore, it appears that EPLBD alone could permanently disrupt sphincter function and cause long-term adverse events in the same manner as EST. However, further study is needed to provide related data that demonstrate it. In addition, a higher incidence of cholangitis without stone recurrence was observed in the EST group than in the EPLBD alone group. We thought it might be related to sphincterotomy stenosis. Because dilatation of the bile duct was detected by MRCP for these patients, dilated CBD may promote bile stasis and bacterial contamination, which play essential roles in a mechanism of cholangitis.

In the present study, multivariate analysis showed that ML was the key factor accounting for the increased incidence of late biliary complications which is in line with the previous study [37]. When CBD stones were not removed, ML was required, which raises the possibility that residual stone fragments, undetectable by cholangiography, might have acted as nidi for stone formation. So we recommend that a regular follow-up be improved for these high-risk patients.

Some limitations of our study must be highlighted: the retrospective design which was conducted at a single center could have resulted in a selection bias; future randomized controlled trials and multicenter study are expected in order to provide the optimal endoscopic therapy for these patients.

In conclusion, as an alternative to EST, EPLBD alone has similar efficacy and safety for treatment of CBD stones. During long-term follow-up, patients who underwent EPLBD alone may have fewer late biliary complications compared with those after EST. In addition, ML may be an independent risk factor for late biliary complications.

## Figures and Tables

**Table 1:** Baseline characteristics of the study patients who underwent EPLBD and EST.

	EST	EPLBD	*P* value
	(*n* = 60)	(*n* = 161)	
Age, mean(SD), years	67.0 (14.6)	70.1 (14.6)	0.17
Sex, male/female	32/28	76/85	0.45
Diameter of CBD (mm), mean (SD)	14.9 (3.3)	14.9 (2.8)	0.85
Number of CBDS, mean (SD)	1.2 (0.6)	1.3 (1.2)	0.58
Maximum stone size (mm), mean (SD)	12.1 (2.8)	12.7 (3.6)	0.2
Periampullary diverticulum, *n* (%)	14 (23.3%)	85 (52.8%)	0.01
Status of gallbladder			
Previous cholecystectomy, *n* (%)	29 (48.3%)	67 (41.6%)	0.45
Cholecystectomy after ERCP, *n* (%)	7 (11.7%)	14 (8.7%)	0.61
Gallbladder with stones in situ, *n* (%)	7 (11.7%)	35 (21.7%)	0.12
Gallbladder without stones in situ, *n* (%)	17 (28.3%)	45 (28.1%)	>0.99

The values are shown in mean ± standard deviation (SD). EPLBD: endoscopic papillary large balloon dilation; EST: endoscopic sphincterotomy; CBD: common bile duct; ERCP: endoscopic retrograde cholangiopancreatography; CBDS: common bile duct stone.

**Table 2:** Comparison of procedural details between the EPLBD group and the EST group.

	EST	EPLBD	*P* value
	(*n* = 60)	(*n* = 161)	
Complete stone removal, *n* (%)	60 (100%)	160 (99.4%)	0.54
In first session	59 (98.3%)	158 (98.8%)	0.81
In second session	1 (1.7%)	2 (1.2%)	
Number of mechanical lithotripsy, *n* (%)	17 (28.3%)	29 (18.0%)	0.1
Method of stone removal, *n* (%)			
Retrieval basket	26 (43.3%)	75 (46.9%)	0.65
Balloon catheter	9 (15%)	19 (11.9)	0.51
Retrieval basket and balloon catheter	22 (36.7%)	61 (38.1%)	0.88
Mechanical lithotripsy basket	3 (5.0%)	5 (3.1%)	0.69
Hospital days, mean (SD)	7.4 (2.5)	7.8 (3.3)	0.49

**Table 3:** Comparison of the adverse events between the EST and EPLBD groups.

	EST	EPLBD	*P* value
	(*n* = 60)	(*n* = 161)	
Pancreatitis	2 (3.3%)	8 (5.0%)	0.73
Mild	2	7	
Moderate	0	1	
Severe	0	0	
Hemorrhage	0	0	—
Cholangitis	2 (3.3%)	3 (1.9%)	0.62
Perforation	0	0	—
Overall adverse events	4 (6.7%)	11 (6.8%)	1.00

**Table 4:** Comparison of the late biliary complications during follow-up between the EST and EPLBD groups.

	EST	EPLBD	*P* value
	(*n* = 59)	(*n* = 157)	
Duration of follow-up (months), mean (SD)	74.5 (20.2)	71.6 (24.5)	0.42
Recurrent bile duct stones, *n* (%)	8 (13.6%)	16 (10.2%)	0.47
Cholangitis without stone recurrence, *n* (%)	3 (5.1%)	0	0.02
Total biliary complications, *n* (%)	11 (18.6%)	16 (10.2%)	0.11

**Table 5:** Univariate and multivariate analyses for risk factors of the late biliary complications.

	Late complication	Without late complication	*P* value
	*n* = 26	*n* = 190	
Age (y)	67.0 (16.4)	69.6 (14.5)	0.398
Sex, male/female	12/14	94/96	0.836
Diameter of CBD (mm), mean (SD)	15.7 (3.2)	14.8 (2.9)	0.140
Number of CBDS, mean (SD)	1.3 (0.7)	1.3 (1.1)	0.997
Maximum stone size (mm), mean (SD)	12.2 (2.6)	12.6 (3.5)	0.570
Periampullary diverticulum, *n* (%)	14 (53.8%)	91 (47.9%)	0.677
Mechanical lithotripsy, *n* (%)	11 (42.3%)	34 (17.9)	0.008
Previous cholecystectomy, *n* (%)	15 (57.7%)	100 (52.6%)	0.679
Gallbladder with stones in situ, *n* (%)	7 (26.9%)	34 (17.9%)	0.288
Gallbladder without stones in situ, *n* (%)	4 (15.4%)	56 (29.6%)	0.164
	Odds ratio	95% Confidence interval	*P* value
Mechanical lithotripsy	2.815	1.148–6.902	0.024

## Data Availability

The data used to support the findings of this study are restricted by the institutional review board of Ruijin Hospital, Affiliated to Shanghai Jiao Tong University School of Medicine, in order to protect the patient privacy.

## Abbreviations

EPLBD: Endoscopic papillary large balloon dilation
EST: Endoscopic sphincterotomy
CBD: Common bile duct
CT: Computed tomography
MRCP: Magnetic resonance cholangiopancreatography
ERCP: Endoscopic retrograde cholangiopancreatography
ML: Mechanical lithotripsy.
